# Hypomethylation of the *ENPP3* promoter region contributes to the occurrence and development of ovarian endometriosis via the AKT/mTOR/4EBP1 signaling pathway

**DOI:** 10.17305/bb.2023.9989

**Published:** 2024-08-01

**Authors:** Yuzhen Qin, Yishuai Li, Yali Hao, Yan Li, Shan Kang

**Affiliations:** 1 Department of Obstetrics, The Fourth Hospital of Hebei Medical University, Shijiazhuang, China; 2 Department of Thoracic Surgery, Hebei Chest Hospital, Shijiazhuang, China; 3 Department of Reproductive Medicine, The Fourth Hospital of Hebei Medical University, Shijiazhuang, China; 4 Department of Molecular Biology, The Fourth Hospital of Hebei Medical University, Shijiazhuang, China; 5 Department of Gynecology, The Fourth Hospital of Hebei Medical University, Shijiazhuang, China

**Keywords:** ENPP3, endometriosis (EMs), DNA methylation, epigenetic, transcriptome sequencing analysis

## Abstract

Growing evidence indicates that aberrant methylation is pivotal in the development and progression of endometriosis (EMs). This study explores the relationship between abnormal methylation of the *ENPP3* promoter and the pathogenesis of ovarian EMs, focusing on its regulatory effect on ENPP3 expression. We analyzed the methylation levels of *ENPP3* in ectopic endometrial tissues from ovarian EMs patients and in normal endometrial tissues from women without EMs. The expression and distribution of *ENPP3* were evaluated using RT-qPCR and immunohistochemistry. Transwell assays were conducted to examine the impact of ENPP3 overexpression on the migratory and invasive capabilities of endometrial stromal cells. Our results demonstrated significantly reduced methylation levels at the CpG sites of the *ENPP3* promoter region in ectopic endometrial tissues compared to normal endometrial tissues. RT-qPCR findings revealed a marked increase in *ENPP3* expression in ovarian EMs tissues relative to endometrial tissues from patients without EMs, and this upregulation was negatively correlated with the methylation levels of the *ENPP3* promoter region. Immunohistochemical analyses confirmed elevated ENPP3 expression in the glandular epithelial cells and stroma of ovarian EMs tissues. Furthermore, in vitro experiments showed that overexpressed ENPP3 notably intensified the invasion and migration of endometrial stromal cells. Transcriptome sequencing and functional analyses indicated that the increased ENPP3 expression activated the AKT/mTOR/4EBP1 signaling pathway. In summary, the study suggests that hypomethylation in the *ENPP3* promoter region may contribute to the initiation and advancement of ovarian EMs by activating the AKT/mTOR/4EBP1 pathway, supporting the theory that EMs might be an epigenetically regulated disorder.

## Introduction

Endometriosis (EMs) is a common inflammatory hormone-dependent condition, affecting approximately 10% of women of reproductive age globally [1]. Its primary characteristics include the occurrence of endometrial glands and stroma outside the uterine cavity, which often results in severe pelvic pain and infertility [2]. Despite various hypotheses, such as coelomic metaplasia, vascular and lymphatic metastatic spread, retrograde menstruation, and altered inflammatory response, the exact pathogenesis of EMs remains unclear [3]. Recent evidence increasingly suggests that abnormal gene expression, linked to DNA methylation, plays a significant role in the emergence and progression of EMs.

DNA methylation, particularly in gene promoter regions, plays a crucial role in gene transcription regulation and has emerged as a key factor in the pathology of EMs [4]. Numerous studies demonstrated that aberrant DNA methylation in these regions contributes to the pathogenesis and progression of EMs, affecting genes, such as *ERβ*, *TWIST*, *RASSF1A*, and *CDH1* [5–8]. In line with these findings, our research has also identified significant hypomethylation of *ENPP3* in both ectopic and eutopic endometrial tissues [9].

ENPP3 is part of ectonucleotide pyrophosphatases/phosphodiesterase (ENPP) family and plays a role in various physiological processes. It is particularly noted for promoting cell motility and migration in tumors [10, 11]. Increased levels of ENPP3 have been observed in conditions, such as clear cell renal cell carcinoma, bile duct carcinoma, and colorectal cancer, positioning ENPP3 as a potential tumor marker [10, 12, 13]. Although EMs is benign, it shares several characteristics with malignant tumors, including hyperplasia, invasion, metastasis, recurrence, and the potential for malignant transformation [14]. Therefore, we hypothesized that hypomethylation of ENPP3 methylation may be involved in the pathogenesis of EMs by upregulating ENPP3 expression.

In our study, we analyzed the methylation status of the *ENPP3* gene promoter region and evaluated ENPP3 expression in ectopic endometrial tissues from patients with ovarian EMs, as well as in normal endometrial tissues from women without EMs. Additionally, we conducted in vitro experiments to induce ENPP3 overexpression in endometrial stromal cells, aiming to investigate its effects on the migration and invasion of these cells. We also performed transcriptome sequencing and functional analysis to identify significantly enriched pathways related to these processes.

## Materials and methods

### Patients and samples

This is a retrospective case-control study, including 70 patients with ovarian EMs (case group) and 60 without EMs (control group). Ectopic endometrium was collected from patients with ovarian EMs who underwent laparoscopic surgery and did not receive any hormonal treatment before surgery. The control endometrium was obtained from patients without EMs who underwent total hysterectomy for cervical intraepithelial neoplasia III. All participants were premenopausal and underwent surgical treatment during the secretory phase of the menstrual cycle (according to the last menstrual time and pathological characteristics). The severity of EMs was rated using the revised American Society for Reproductive Medicine (ASRM) criteria. All samples were used to perform real-time quantitative polymerase chain reaction (RT-qPCR) and immunohistochemistry (IHC). The final analysis of DNA methylation group consisted of 31 cases and 30 controls. There was no statistically significant difference in age distribution between two groups (*P* > 0.05, Table 1).

**Table 1:** Comparison of age between case and control groups

	**Samples for DNA methylation analysis**		**Samples for RT-qPCR and IHC assay**	
	**Control**	**Case**	**Control**	**Case**
*n*	30	31	60	70
Range of age (years)	24–47	32–51	24–49	32–53
Age, mean ± SD (years)	36.57± 5.90	38.24± 6.30	36.77± 6.41	39.94± 6.47
*P* value	0.13		0.18	

RT-qPCR: Real-time quantitative polymerase chain reaction; IHC: Immunohistochemistry.

### Genomic DNA extraction

With Wizard Genomic DNA Purification Kit (Promega, Madison, WI, USA), the genomic DNA was extracted from the tissues. With a UV spectrophotometer (NanoDrop 2000; Thermo Fisher Scientific, Wilmington, DE, USA), the concentration and purity of DNA were evaluated by the absorbance at 260 nm and 280 nm.

### DNA methylation analysis

Quantitative methylation analysis of the DNA fragments of *ENPP3* gene was performed by using Agena MAssARRAY platform (CapitalBio, Beijing, China). A total of 1 µg of genomic DNA from each sample was converted with sodium bisulfite using the EZ DNA methylation kit (Zymo Research, Orange, CA, USA) and the modified DNA was amplified by PCR. PCR primers for ENPP3 gene were designed with Epidesigner (http://www.epidesigner.com, Sequenom Inc., San Diego, CA, USA). Each forward primer was tagged with a 10 mer (5’-AGGAAGAGAG-3’) to balance the PCR, and each reverse primer had a T7-promoter tag (5’-CAGTAATACGACTCACTATAGGGAGAAGGCT-3’) for transcription in vitro (Table 2). After the treatment by shrimp alkaline phosphatase (MassCLEAVE Kit, Agena) and transcription in vitro, the PCR products were treated according to the standardized protocol of SequenomEpiTyper Assay and further cleaned by resin and dispensed to the 384 SpectroCHIP by Nanodispenser. The chips were read by the Agena Mass Spectrometer system and spectra methylation ratios were generated by Epityper v1.2 software (Agena Bioscience, San Diego, CA, USA).

**Table 2:** Primer sequences for methylation analysis and RT-qPCR

**Primers**	**Sequences**
*Methylation analysis*	
Left primer plus tag	5’-aggaagagagTAGTGATTGGGTAATTTGGAGAGAG-3’
Right primer plus tag	5’-cagtaatacgactcactatagggagaaggctCCTTAATTTACTCATCTAAAAACAAAAA-3’
*RT-qPCR*	
*ENPP3*	
Forward primer	5’-CCGCATCCGAGCTCATAATATA-3’
Reverse primer	5’-CTTTGGCAAATCAGGAGTCAAA-3’
*GAPDH*	
Forward primer	5’-ACCACAGTCCATGCCATCAC-3’
Reverse primer	5’-TCCACCACCCTGTTGCTGTA-3’

RT-qPCR: Real-time quantitative polymerase chain reaction.

### RNA extraction and RT-qPCR

Total RNA was extracted from the samples with TRIzol (Generay Biotech Co., Ltd., Shanghai, China). Total cDNA was synthesized by using the Revert Aid First Strand cDNA Synthesis Kit (Thermo Scientific, USA) according to the manual. The primers for ENPP3 and GAPDH were designed by Sangon Biotech Co., Ltd. (Shanghai, China) and the sequences are displayed in Table 2. The qPCR experiments were performed in Agilent M x 3000P Real-time PCR system using the QuantiNovaTM SYBR^®^ Green PCR Kit (Qiagen, Hilden, Germany). The reaction was set at 95 ^∘^C for 5 min for pre-denaturation, followed by 95 ^∘^C for 15 s, then 58 ^∘^C for 30 s, and, finally, 72 ^∘^C for 25 s, repeating 40 cycles. The dissolution curve program setup started with 95 ^∘^C for 15 s, and was followed by 60 ^∘^C for 1 min, and then 95 ^∘^C for 30 s. The relative expression of ENPP3 mRNA was calculated with the 2^−ΔΔCt^ method and the experiments were repeated three times.

### Immunohistochemistry (IHC)

Sections thick 4 µm were dewaxed in xylene and dehydrated through a graded series of ethanol. Antigen was retrieved by heating in sodium citrate (pH 6.0) for 10 min. After washing in PBS, slides were blocked with 3% hydrogen peroxide blocking serum for 15 min at room temperature. After blocking endogenous peroxidase and nonspecific binding, the sections were incubated overnight at 4 ^∘^C with primary antibody (Anti-ENPP3/B10 antibody, Abcam, ab233777, Cambridge, UK; dilution 1:500). After three washes in PBS, the sections were incubated with secondary antibody (goat anti-rabbit IgG, Abcam, ab97080, Cambridge, UK; dilution 1:500) for 45 min at room temperature. After the sections were washed in PBS, they were incubated with DAB reagent and counterstained with hematoxylin. Negative control sections were incubated with PBS instead of primary antibody. The sections were independently examined by two pathologists, who were blinded to the clinicopathological data. Specimens were scored according to the intensity of the dye color and the number of positive staining area: 1 (light yellow), 2 (light brown), and 3 (brown), whilst the number of positive cells was graded as 0, 1 (1%–25%), 2 (26%–50%), 3 (51%–75%), and 4 (>75%). When the two grades were added, specimens were assigned to one of the 4 levels: 0–1 score (−), 2–4 scores (+), 5–8 scores (++), more than 9 scores (+++).

### Cell lines and transfection

The primary human endometrial stromal cells (HESCs) were isolated and cultured as described previously [15]. The HESCs were transfected by the ENPP3 expression plasmid (EX-U0254-M98) or empty vector (EX-NEG-M98) purchased from IGenebio (Guangzhou, China). Transfections were performed using Lipofectamine^TM^ 2000 transfection reagent (Invitrogen, MA, USA) according to the manufacturer’s protocol. RT-qPCR and Western blot analyses were used to quantify the mRNA and protein expression of ENPP3 48 h after transfection.

### Western blot analysis

HESCs were collected 48 h after transfection to perform Western blot analysis. The cells were lysed by RIPA lysis buffer (Solarbio, China), and the total protein content was determined by BCA assay (Solarbio, China). Equal amounts of protein (30 µg) were subjected to 10% SDS-PAGE and transferred onto polyvinylidene difluoride (PVDF) membranes (Bio-Rad Laboratories, Inc., CA, USA). Membranes were blocked with 5% nonfat powdered milk and incubated with primary antibodies (mouse polyclonal antibody for ENPP3 (Abcam, ab233777, Cambridge, UK; dilution 1:2000), ACTIN (Servicebio, GB12001, Wuhan, China; dilution 1:2000) overnight at 4 ^∘^C. Upon washing with PBST, membranes were incubated with anti-rabbit secondary antibody (Servicebio, GB 23303, Wuhan, China; dilution 1:5000) for 1 h at room temperature. The protein bands were visualized by using ECL (NCM Biotech, China) and membranes were scanned by Odyssey infrared imaging system (LI-COR Biosciences, Lincoln, NE, USA).

### Transwell assay

Cell invasion was examined with transwell assay (Corning, Inc., NY, USA). The upper chamber was covered with a matrigel solution and dried overnight at 37 ^∘^C. Afterward, the cells (1 × 10^5^ per 200 µL) were inoculated in the upper chamber with a serum-free medium. To the lower chamber, 800 µL of complete medium was added. After a 48 h-long incubation at 37 ^∘^C, the transwell chambers were washed twice with PBS and cells were fixed with 4% paraformaldehyde for 30 min at room temperature. After applying crystal violet solution for 20 min, the matrigel and cells from the top surface of the membrane were gently removed with a cotton swab. The number of cells that infiltrated the transwell chamber membrane was counted under a microscope. The experiment was repeated three times. The migration assay was the same as the transwell invasion experiment without the addition of matrix glue.

### Transcriptome sequencing analysis

Transcriptome sequencing analysis was performed on HESCs cells transfected by EX-U0254-m98 plasmid (ENPP3-plasmid group) and empty plasmid (NC group) to obtain differentially expressed genes (DEGs) between the two groups. Total RNA was extracted from the samples with TRIzol (Generay Biotech Co., Ltd., Shanghai, China). RNA-sequencing was performed on Illumina system. Raw data was preprocessed for batch correction and normalization by using R software (version 3.6.1). Clean reads were obtained by removing low quality reads containing adapter and ploy-N from raw data. HISAT2 (v2.0.5) was used to align the clean reads with the human reference genome. DESeq2 was used to identify DEGs with |log_2_(Fold Change)|>1 and *P* < 0.05. Then, KEGG analysis was performed to screen out the enrichment pathways of great significance. Finally, protein levels of the important molecules from pathways were analyzed using the Western blot assay.

### Ethical statement

This study was approved by the Ethics Committee of the Fourth Hospital of Hebei Medical University (2021ky316). The procedures used in this study adhere to the tenets of the Declaration of Helsinki. All subjects gave their written informed consent. All the paraffin-embedded tissues used for ENPP3 IHC in this study were collected from the Fourth Hospital of Hebei Medical University in China.

### Statistical analysis

Statistical analysis was performed by the SPSS 24.0 (Chicago, IL, USA) and GraphPad Prism 8.0 software. Results were presented as mean ± SD/SEM of at least three independent experiments. Student’s *t*-test and non-parametric test were used to assess the differences between two groups. Spearman’s correlation analysis was used to evaluate the association between the two variables. The differences were considered significant at *P* < 0.05 for two-sided analyses.

## Results

### Hypomethylation of ENPP3 promoter region in EMs

**Figure 1.**
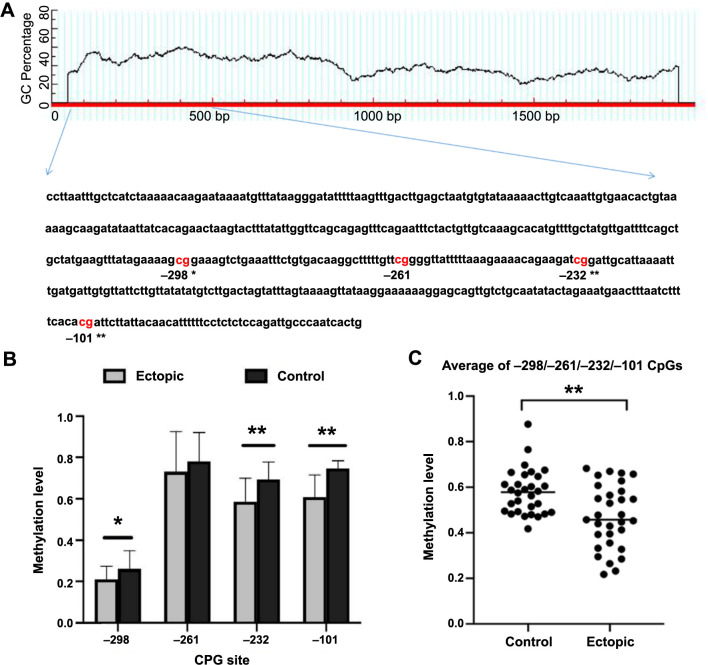
**The promoter region of *ENPP3* was significantly hypomethylated in the ectopic endometrium of patients with ovarian EMs.** (A) The measured fragment in the ENPP3 promoter region, including four CpG sites (−101, −232, −261, and −298); (B) The ENPP3 methylation level of each detectable CpG site in the ectopic and control endometrial tissues, data are represented as mean ± SD; (C) The average ENPP3 methylation level of fragment in the ectopic was significantly lower compared to the control endometrial tissues, data are represented as mean ± SEM. ^*^P < 0.05, ^**^P < 0.01. EMs: Endometriosis.

Four CpG sites (−298, –61, −232, and −101) were detected by MassARRAY analysis (Figure 1A). The methylation levels of the −298/−232/−101 CpGs were pronouncedly lower in the ectopic endometrium of patients with ovarian EMs compared to the control group (Figure 1B). The average methylation levels of the four detectable CpGs were significantly lower in the ovarian EMs tissues compared to the endometrial tissues from patients without EMs (0.58 ± 0.09 and 0.47 ± 0.13, respectively) (*P* < 0.001) (Figure 1C).

### ENPP3 expression in ovarian EMs tissues

**Figure 2.**
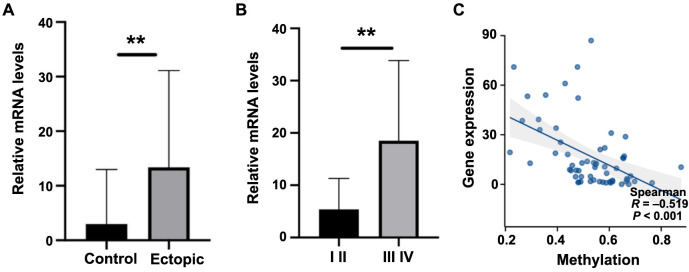
**The expression of ENPP3 in ovarian EMs tissues.** (A) The relative mRNA level of ENPP3 in the ectopic tissues was significantly increased compared to the control endometrial tissues, data are represented as mean ± SD; (B) ENPP3 was significantly upregulated in the late stage compared to the early stage of EMs, data are represented as mean ± SEM; (C) The mRNA expression level of ENPP3 was significantly negatively correlated with the methylation level of ENPP3 in the promoter region. ^**^P < 0.01. EMs: Endometriosis.

**Figure 3.**
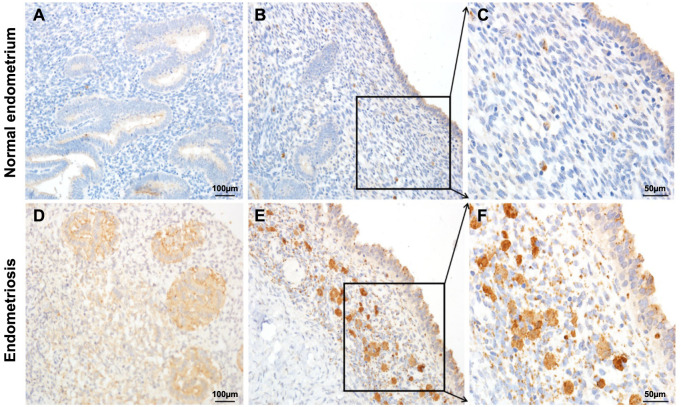
**Representative immunohistochemical staining of ENPP3 in the endometrial tissues.** (A–C) In the endometrial tissues from patients without EMs, ENPP3 was mainly expressed in the cytoplasm of glandular epithelial cells and less expressed in the stroma; (D–F) In ovarian EMs tissues, strong expression could be seen both in glandular epithelial cells and stroma. EMs: Endometriosis.

The RT-qPCR results indicated that *ENPP3* was significantly upregulated in the ectopic endometrium tissues (*P* < 0.01) (Figure 2A). In addition, *ENPP3* was significantly upregulated in the late stage (stage III-IV, *n* ═ 33, 18.53 ± 3.001) than early stage (stage I-II, *n* ═ 37 5.4 ± 1.081) of EMs (*P* < 0.01, Figure 2B). Spearman’s correlation analysis showed that the mRNA expression level of *ENPP3* was significantly negatively correlated with the methylation level of *ENPP3* in promoter region (average of the −298/−261/−232/−101 CpGs; *r* ═ 0.519, *P* < 0.001, Figure 2C). To evaluate the protein levels of ENPP3, the paraffin pathological sections of the two groups were examined by IHC. Immunohistochemical staining showed that ENPP3 was mainly expressed in the cytoplasm of endometrial epithelial cells and less expressed in the stroma of endometrial tissues from patients without EMs (Figure 3A–3C). However, in patients with EMs, there was a higher expression both in glandular epithelial cells and stroma in ovarian EMs tissues (Figure 3D–3F, Table 3).

**Table 3:** ENPP3 protein expression in glandular epithelium and stroma of ectopic endometrium (*n* ═ 70) and normal endometrium (*n* ═ 60)

	**Groups**	**IHC grades**				***P* value**
		**−**	**+**	**++**	**+++**	
Glandular epithelium	Ectopic endometrium	0	24	39	7	<0.001
	Normal endometrium	2	52	6	0	
Stroma	Ectopic endometrium	2	17	33	18	<0.001
	Normal endometrium	52	8	0	0	

IHC: Immunohistochemistry.

### Effect of EX-U0254-m98 on ENPP3 expression in cell lines

**Figure 4.**
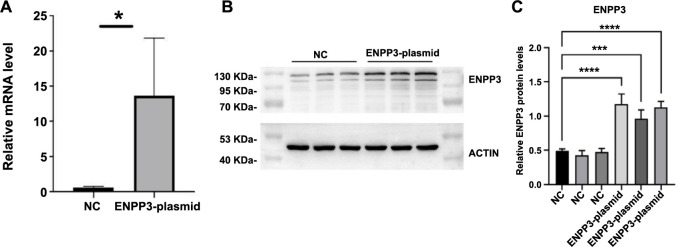
**Effect of ENPP3 overexpression plasmid (EX-U0254-m98) on ENPP3 expression.** (A) Relative mRNA levels of ENPP3 in the EX-U0254-m98 transfection group (ENPP3-plasmid group) and empty vector transfection group (NC); (B and C) Protein expression of ENPP3 in the EX-U0254-m98 transfection group (ENPP3-plasmid group) and empty vector transfection group (NC). Data are represented as mean ± SD. ^*^P < 0.05, ^***^P < 0.001, ^****^P < 0.0001.

HESCs were transfected with the EX-U0254-m98 plasmid (ENPP3-plasmid group) and an empty plasmid, respectively. RT-qPCR analysis revealed that the expression of *ENPP3* was significantly higher in the ENPP3-plasmid group compared to the group transfected with the empty vector (*P* < 0.05, Figure 4A). Similarly, Western Blot results indicated that the protein levels of ENPP3 were significantly elevated in the ENPP3-plasmid group compared to the empty vector group (*P* < 0.05, Figure 4B and 4C).

### Overexpression of ENPP3 promoted cell migration and invasion

**Figure 5.**
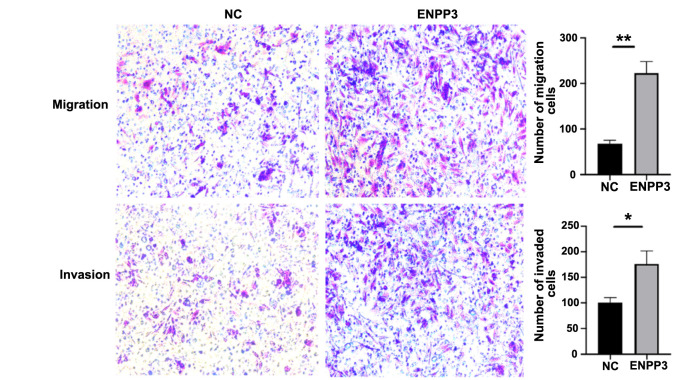
**Overexpression of ENPP3 promoted cell migratory and invasive capabilities.** The migratory and invasive capabilities of the HESCs were increased in the ENPP3-plasmid group compared to the empty vector transfection group (NC). The bar graphs represent the average number of migrated and invaded cells on the underside of the membrane. Data are representative of three experiments and represented as mean ± SD. ^**^P < 0.01. HESC: Human endometrial stromal cell.

The migration and invasion capabilities of cells were evaluated using a Transwell assay. The findings revealed that overexpression of ENPP3 significantly enhanced both the migration (*P* < 0.01) and invasion (*P* < 0.01) of HESCs (Figure 5).

**Figure 6.**
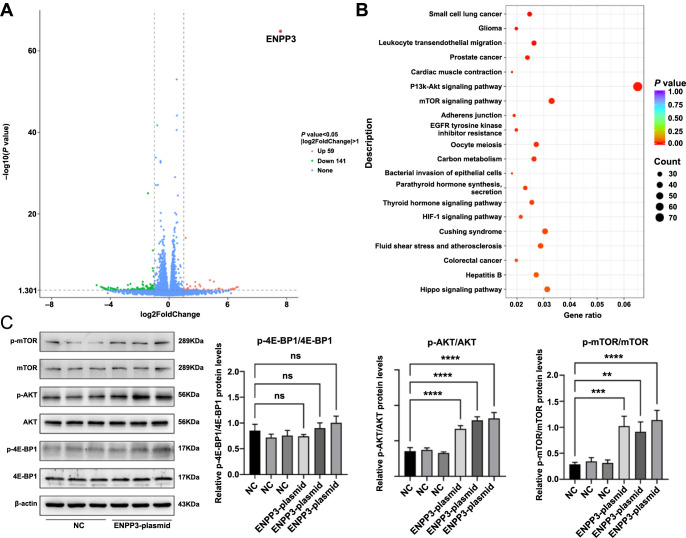
**Overexpression of ENPP3 activated the AKT/mTOR/4EBP1 signaling pathway.** (A) Volcano plot for differentially expressed genes, highlighting highly expressed ENPP3 (top right corner); (B) Significant KEGG pathway terms for differentially expressed genes; (C) Protein and phosphorylated protein levels for the AKT/mTOR/4EBP1 signaling pathway and its downstream molecules detected by western blotting. Data are represented as mean ± SD. ^*^P < 0.05, ^**^P < 0.01, ^***^P < 0.001, ^****^P < 0.0001. HESC: Human endometrial stromal cells.

### Overexpression of ENPP3 activated the AKT/mTOR/4EBP1 signaling pathway

Transcriptome sequencing identified 200 DEGs, comprising 59 upregulated and 141 downregulated genes (Figure 6A). Functional analysis suggested significant involvement of the PI3K-AKT and mTOR signaling pathways (Figure 6B). Western Blot analysis revealed that overexpression of ENPP3 could activate the AKT/mTOR/4EBP1 signaling pathway (Figure 6C). While the total protein levels of 4EBP1 and p-4EBP1 exhibited an upward trend, this was not statistically significant. However, the phosphorylation level of 4EBP1 was significantly higher in the ENPP3-plasmid group (*P* < 0.05, data not shown).

## Discussion

To our knowledge, this study is the first to document hypomethylation of the *ENPP3* promoter region in ectopic endometrial tissues, a condition inversely correlated with elevated *ENPP3* gene expression. This finding aligns with the role of *ENPP3* in tumors and is mirrored in EMs, where we demonstrated that increased *ENPP3* expression augments the invasion and migration of endometrial epithelial cells.

Recent evidence has suggested that EMs may be an epigenetically regulated disease [16]. Aberrant DNA methylation appears to play a role in the onset and progression of EMs by regulating the expression of specific genes such as *ESR1*, *COX-2*, *HOXA10*, and *NR5A1* [17, 18]. Consistent with prior studies, the results of our study support the idea that EMs may be an epigenetically regulated disease. We observed markedly reduced methylation levels in the *ENPP3* promoter region within ectopic endometrial tissue. Notably, the CpG sites spanning from −53 to −444 upstream of the *ENPP3* gene might serve as transcription factor binding sites, including for *FOXA1*, *AR*, and *ESR1* (source: http://jaspar.genereg.net/). These hypomethylated CpG sites may impact the binding ability of transcription factors, potentially leading to the transcriptional activation of *ENPP3*. Indeed, ENPP3 mRNA expression was significantly upregulated in ectopic endometrial tissue and inversely correlated with the methylation levels of the *ENPP3* promoter region.

The expression of ENPP3 is suggested to dynamically changes during the female menstrual cycle, peaking during the secretory phase [19]. The ENPP3 protein is specifically localized on the apical surface of endometrial epithelial cells and glandular secretions [19, 20]. A previous research by Boggavarapu et al. [21] indicated the potential use of ENPP3 as a non-invasive test for endometrial receptivity. However, to date, there have been only two reports on the expression of ENPP3 in EMs and the results have not been entirely consistent [22, 23]. Thus the clinical significance of *ENPP3* methylation in EMs remains to be fully elucidated. In this study, we carefully selected endometrial tissue from both 70 patients with EMs and 60 patients without EMs during the secretory phase to minimize the bias caused by the menstrual cycle. Our findings indicate a noticeable increase in ENPP3 expression at both mRNA and protein levels in ovarian EMs tissues. In endometrial tissues from patients without EMs, ENPP3 was primarily expressed in endometrial epithelial cells but usually absent in the stroma. Only 13% (8/60) showed weak ENPP3 staining in the stroma. In contrast, in patients with EMs, intense expression was observed in both endometrial epithelial cells and stroma.

ENPP3 has been reported to facilitate tumor cell invasion [24]. Similar effects are evident in other processes and diseases, albeit variably across different tumor areas. For instance, a study showed that *ENPP3* upregulation during embryo implantation could enhance trophoblastic cell invasion [24]. Additionally, ENPP3 was found to regulate the proliferation and migration of vascular smooth muscle cells in post-PTA restenosis in lower extremity arteries, mediated by GRIA2 regulation [25]. To investigate the impact of ENPP3 elevation on endometrial cells, we constructed an ENPP3 overexpression plasmid and transfected it into HESCs. Transwell assays indicated that ENPP3 enhancement significantly enhanced the migratory and invasive capabilities of endometrial stromal cells, suggesting that hypomethylation-driven ENPP3 expression increase is implicated in ovarian EMs. This research marks the first demonstration of ENPP3 upregulation significantly boosting endometrial stromal cell migration and invasion.

The PI3K/AKT/mTOR pathway is known to be aberrantly activated in EMs [26]. Previous studies have indicated that heightened PI3K/AKT pathway activity impairs the decidualization of stromal cells in EMs [27], with mTOR signaling activation contributing to disease initiation by fostering ectopic lesion development [28]. *4EBP1*, a principal downstream effector of the mTOR signaling pathway, positions the PI3K/AKT/mTOR pathway as a potential therapeutic target in EMs [26]. Our transcriptome sequencing analysis revealed that ENPP3 overexpression predominantly influenced the PI3K-AKT and mTOR signaling pathways. Western blot analysis further demonstrated that AKT/mTOR/4EBP1 signaling pathway-related factors were upregulated in the ENPP3-plasmid group, indicating activation of the AKT/mTOR/4EBP1 signaling pathway by ENPP3 overexpression. These results suggest that ENPP3 elevation might promote EMs through the activation of the AKT/mTOR/4EBP1 signaling pathway.

Despite the discovery of ENPP3’s role in enhancing the motility and invasiveness in 1999, the precise molecular mechanisms remain elusive [24]. Further multidisciplinary research is essential to elucidate ENPP3’s specific molecular function in EMs. One limitation of our study was the lack of simultaneous assessment of ENPP3 expression in peripheral blood. If serum ENPP3 expression correlates positively with tissue expression, it could become a molecular marker for EMs. Moreover, an antibody-drug conjugate (ADC) targeting ENPP3 is undergoing phase II clinical trials for advanced renal cell carcinoma treatment [29], offering a promising therapeutic approach for EMs. Thus, further research is crucial for the clinical diagnosis and treatment of EMs.

## Conclusion

In summary, our findings have identified hypomethylation in the *ENPP3* promoter region and noted elevated ENPP3 mRNA and protein levels in ectopic endometrial tissues. The hypomethylation in the *ENPP3* promoter region negatively regulates ENPP3 expression, potentially contributing to EMs’ onset and progression. Our transcriptome sequencing and functional analysis imply that ENPP3 overexpression may promote EMs by activating the AKT/mTOR/4EBP1 signaling pathway. This adds to the evidence that EMs might be an epigenetically regulated disorder.
